# Miscarriage hospitalisations: a national population-based study of incidence and outcomes, 2005–2016

**DOI:** 10.1186/s12978-019-0720-y

**Published:** 2019-05-09

**Authors:** Indra San Lazaro Campillo, Sarah Meaney, Keelin O’Donoghue, Paul Corcoran

**Affiliations:** 1 Pregnancy Loss Research Group, The Irish Centre for Fetal and Neonatal Translational Research University College Cork, Cork, Ireland; 2 National Perinatal Epidemiology Centre, Department of Obstetrics and Gynaecology, University College Cork, Cork University Maternity Hospital, 5th floor, Postgraduate Study Room, 5S-30, Wilton, Cork, T12 YE02 Ireland; 3 The Irish Centre for Fetal and Neonatal Translational Research, Department of Obstetrics and Gynaecology, University College Cork, Cork, Ireland; 4 School of Public Health, University College Cork, Cork, Ireland

**Keywords:** Miscarriage, Hospitalisations, Rates, Morbidity

## Abstract

**Background:**

Early miscarriage is one of the most common obstetric causes of maternal morbidity early in pregnancy. However, data concerning non-fatal complications among hospitalisations for early miscarriage are lacking. The aim of this study was to determine whether there were changes in the incidence, management and outcomes of early miscarriage hospitalisations between 2005 and 2016.

**Methods:**

This is a nationwide population-based study of 50,538 hospitalisations with a diagnosis of early miscarriage of all acute maternity hospitals in Ireland. Electronic health records were retrieved using the Hospital In-Patient Enquiry database. Main outcomes include the incidence rates of hospitalisations and management for early miscarriage, and rates of blood transfusion and length of stay over 2 days.

**Results:**

Overall, 50,538 hospitalisations for early miscarriage were identified from 2005 to 2016. The risk of hospitalisation decreased from 70.6 per 1000 deliveries (95% CI 68.4 to 72.8) in 2005 to 49.7 per 1000 deliveries (95% CI 49.7 to 53.3) in 2016; however, the risk of blood transfusion increased over time (ratio: 2.0; 95% CI 1.6 to 2.4). Women of advanced maternal age had a higher risk of hospitalisations. There were less blood transfusions among women who undertook medical treatment (ratio: 0.3; 95% CI 0.1 to 0.5), but they had an increased risk of staying over 2 days at the hospital (ratio: 1.5; 95% CI 1.2 to 1.9) compared to evacuation of retained products of conception.

**Conclusions:**

Hospitalisation rates for early miscarriage decreased over time with an increase in risk of blood transfusion and an extended length of stay at the hospital. Women who underwent medical management did not have as many blood transfusions as those undergoing surgical management. However, they had an increased risk of an extended stay. Research is needed to explore both outpatient and inpatient settings in order to improve the management and care provided.

**Electronic supplementary material:**

The online version of this article (10.1186/s12978-019-0720-y) contains supplementary material, which is available to authorized users.

## Plain English summary

Approximately, 1 out of 4 women will experience an early miscarriage in their reproductive life. Despite the burden of early miscarriage, there is a lack of information regarding trends in incidence rates of hospitalisations and type of management of early miscarriage, but also about the morbidities associated to hospitalisations of early miscarriage. Therefore, the objectives of this study were to explore national trends in incidence rates of hospital admissions for early miscarriage in the Republic of Ireland from January of 2005 to December of 2016, and to estimate morbidity associated with blood transfusion and length of stay over 2 days.

This is a retrospective population-based study using the Hospital In-Patient Enquiry (HIPE). The HIPE is a computer-based system designed to collect demographic, clinical and administrative data on discharges and deaths in the Republic of Ireland. However data from the emergency department and outpatient settings are not available.

Over this period of time there were approximately 50,000 hospitalisations for early miscarriage. Early miscarriage hospitalisations became 19% less common during 2005–2016 but the risk of blood transfusion doubled. The risk of an extended length of stay also increased over the same time period. Women who underwent medical management did not have as many blood transfusions compare to those who had surgical management. However, women who underwent medical treatment had a higher risk of a prolonged stay at the hospital. More research is needed to explore the patterns of care and morbidities associated to hospitalisation in order to improve protocols of management and the care provided for women who miscarry.

## Background

Miscarriage is one of the most common complication in early pregnancy [1–3]. It is clinically classified as either early miscarriage, within 13 weeks of gestation, or late miscarriage, between 13 and before 24 completed weeks of pregnancy [1, 4–6]. Early miscarriage occurs in 10 to 30% of all pregnancies [7, 8] and in 11 to 16% of all clinically recognised pregnancies [9, 10]. Late miscarriage is estimated to occur in less than 1% [11, 12]. Despite the burden of early miscarriage, to our knowledge, no studies have published national trends in incidence rates of hospitalisations for early miscarriage.

The pathways of care for early miscarriage have evolved [13]. Traditionally, the “gold standard treatment” for early miscarriage was surgical uterine evacuation [6]. The introduction and improvement in sensitivity of transvaginal scans (TVS) has helped to diagnose miscarriage early in pregnancy [6, 14]. Furthermore, medical management, using misoprostol [15], and expectant management are acceptable alternative to surgery, which are currently offered to haemodynamically stable patients [13, 16]. However, the optimal management for miscarriage and their associated adverse effects are still being investigated [17].

Hospitalisations during pregnancy are indicative of severe complications [18]. Early miscarriage is associated with less severe complications than ectopic pregnancy [19]; however, heavy bleeding is one of the clinical complications why women who miscarry are admitted to hospital [6, 20]. Moreover, second-trimester miscarriage, while less common, almost always requires inpatient admission and senior obstetric input [11]. Yet, clear and generalised evidence concerning morbidities among hospitalisations for early miscarriage are lacking [21]. Therefore, this study aimed to explore national trends in incidence rates of hospitalisations for early miscarriage, to explore trends in management, and to estimate the associated morbidity of blood transfusion and length of stay.

## Methods

### Study design and data source

A retrospective population-based study was conducted using the Hospital In-Patient Enquiry (HIPE) database. All inpatient admissions for early miscarriage in all public maternity hospital settings in the Republic of Ireland from January 1st 2005 to 31st December 2016 were included. The HIPE is an anonymous national health computer-based system designed to collect demographic, clinical and administrative data on discharges and deaths from all 62 acute hospitals in the Republic of Ireland [22, 23]. Therefore, outpatient data, (i.e. emergengcy department, day patient, early pregnancy assessment units or post-anaesthetic care department) are not available [22, 23]. Outpatient and inpatient data are not linked at a national level in the Republic of Ireland, and therefore, this study was unable to report how many hospitalisation of early miscarriage were referred from emergency department or other outpatient settings. The Economic and Social Research Institute on behalf of the Health Service Executive is the executive organism which administerd and managed the HIPE database [24].

### Population

From 2005, the *10th Revision Australian modification of International Statistical Classification of Disease and Related Health Problems* (ICD-10-AM) and t*he Australian Refined Diagnosis Related Groups* are the coding classification systems of diagnosis used in the HIPE system [22]. All miscarriage hospitalisations within the HIPE dataset were identified using the diagnostic codes for outcome of miscarriage (O03). The unit of analysis was the annual number of delivery discharges within the HIPE dataset using the diagnostic code for outcome of delivery (Z37). According to the ICD-10-AM, miscarriage is defined as the spontaneous expulsion or extraction of the productos of conception by any means, before viability, that being less than 22nd weeks of pregnancy. Miscarriage can be classified as complete miscarriage (i.e. when productos of conception are not evident on ultrasound), but also incomplete miscarriage (i.e. when patient is admitted because of retained products of conception). However, HIPE data does not specify gestational age in single weeks but uses ranges between < 5, 5 to 13, 14 to 19, 20 to 25, 26 to 33 and 34 to 36 completed weeks of gestation. Therefore, our analysis were restricted to early miscarriage, which was defined as a miscarriage before 14 completed weeks.

### Outcomes measures and independent variables

This study included blood transfusion as a complication and length of stay as an indicator of efficiency. Diagnostic codes for blood transfusion were identified using codes within the HIPE dataset (920,600 & 9,206,200 &1,370,601–1,370,603). Length of stay was automatically obtained using the menu of the HIPE database. Hospitalisations with length of stay greater than 2 days were also considered a complication for the purpose of this study.

Demographic and pregnancy-related variables within the HIPE dataset included year of discharge, maternal age (in years) and public or private health insurance. All women who are pregnant and ordinarily resident in the Republic of Ireland are entitled to free maternity care, covering antenatal visits, labour and delivery and postnatal care under the Maternity and Infant Care Scheme [25]. Those inpatient admissions who were treated under the Maternity and Infant Care Scheme were classified as public patients. The only alternative option is to be treated using private health insurance were classified as private patients.

Management for early miscarriage was categorised as surgical and medical treatment. Women were classified as being managed expectantly when neither of the previous procedures codes were identified (i.e. other treatments) or when women had no recorded procedures in HIPE. Surgical treatment included evacuation of retained products of conception. Evacuation of retained products of conception applied when a code for one of the following procedures was recorded: curettage of uterus with (D&C) or without dilatation (3,564,300, 35,640–00 & 35,640–01), suction curettage of uterus (3,564,003 & 3,564,301), dilation and evacuation of uterus (D&E) (35643–03). Medical treatment of early miscarriage involving specific types of prostaglandin E1 (i.e. misoprostol and cervagem) or mifepristone could not be identified as no procedure codes are recorded in HIPE to indicate administration of these drugs. Instead, medical management using codes for prostaglandin, as a general group, or oxytocin were used as the reference medical treatment for early miscarriage. A more detailed description of the principal procedures codes is included in Additional file 1.

In the Republic of Ireland, women with no signs of infection (i.e. vaginal discharge), excessive bleeding, pyrexia or abdominal pain are offered expectant or medical management from the outpatient departments. Surgical management of early miscarriage should be offered to women who make a specific request, who change their mind during the course of conservative or medical management, who have heavy bleeding and/or severe pain, when gestational trophoblastic disease is suspected or when infected intrauterine tissue is present [6].

### Statistical analysis

Hospitalisation incidence rates were estimated using the annual number of inpatient discharges for early miscarriage divided by the annual number of delivieries in the Republic of Ireland over the 12-year period (2005–2016). The crude and adjusted incidence rate ratio of hospitalisation for early miscarriage with 95% confidence intervals (CI) were calculated using univariate and multivariable Poisson regression. All analyses were adjusted by year of discharge, maternal age, public versus private patient and weeks of gestation. The crude and adjusted incidence rate ratio with 95% CI for blood transfusion and length of stay over 2 days was calculated using a multivariable Poisson regression model. Data analysis was performed using Stata software (version 12) and IBM SPSS Statistics for Windows (version 21.0).

## Results

In total, 50,538 hospitalisations for early miscarriage up to 14 completed weeks of gestation and 801,764 deliveries were identified between January 2005 and December 2016. Overall, the rate for hospitalisation of early miscarriage was 63.0/1000 deliveries (95% CI 62.5 to 63.6; Table 1). Approximately 59.0% (*n* = 29,835) of early miscarriages were diagnosed as incomplete miscarriage. Almost 99.4% of all women admitted to maternity hospitals were between 5 to 13 weeks of gestation (*n* = 50,252).

**Table 1:** Incidence rate and incidence rate ratio of hospitalisations for early miscarriage in the Republic of Ireland, 2005–2016

	Deliveries	No of hospitalisations for early miscarriage	Rate^a^ (95% CI)	Crude incidence rate ratio (95% CI)	Adjusted incidence rate ratio ^b^ (95% CI)
All	801,764	50,538	63.0 (62.5–63.6)		
Year					
2005–2008	257,750	17,958	69.7 (68.7–70.7)	1.0 (ref. group)	1.0 (ref. group)
2009–2012	285,751	17,956	62.8 (61.9–63.8)	0.93 (0.91–0.95)	0.85 (0.84–0.88)
2013–2016	258,263	14,624	56.6 (55.7–57.5)	0.95 (0.93–0.97)	0.77 (0.75–0.78)
Maternal Age					
< 25	109,812	6404	58.3 (56.9–59.7)	1.0 (ref. group)	1.0 (ref. group)
25–29	177,647	9071	51.1 (50.0–52.1)	0.60 (0.58–0.62)	0.61 (0.59–0.63)
30–34	281,961	14,697	52.1 (51.3–53.0)	0.90 (0.87–0.92)	1.10 (1.02–1.10)
35–39	191,970	14,250	74.2 (73.0–75.4)	1.27 (1.24–1.31)	1.60 (1.55–1.65)
40+	40,374	6116	151.5 (147.7–155.3)	2.60 (2.51–2.69)	3.34 (3.22–3.45)
Health insurance					
Private	200,014	8951	44.8 (43.8–45.7)	1.0 (ref. group)	1.0 (ref. group)
Public	601,750	41,587	69.1 (68.4–69.8)	1.38 (1.35–1.41)	1.87 (1.83–1.92)

^a^Rate per 1000 deliveries; ^b^ Adjusted incidence rate ratio from multivariable analysis including all variables in the table

**Fig. 1:**
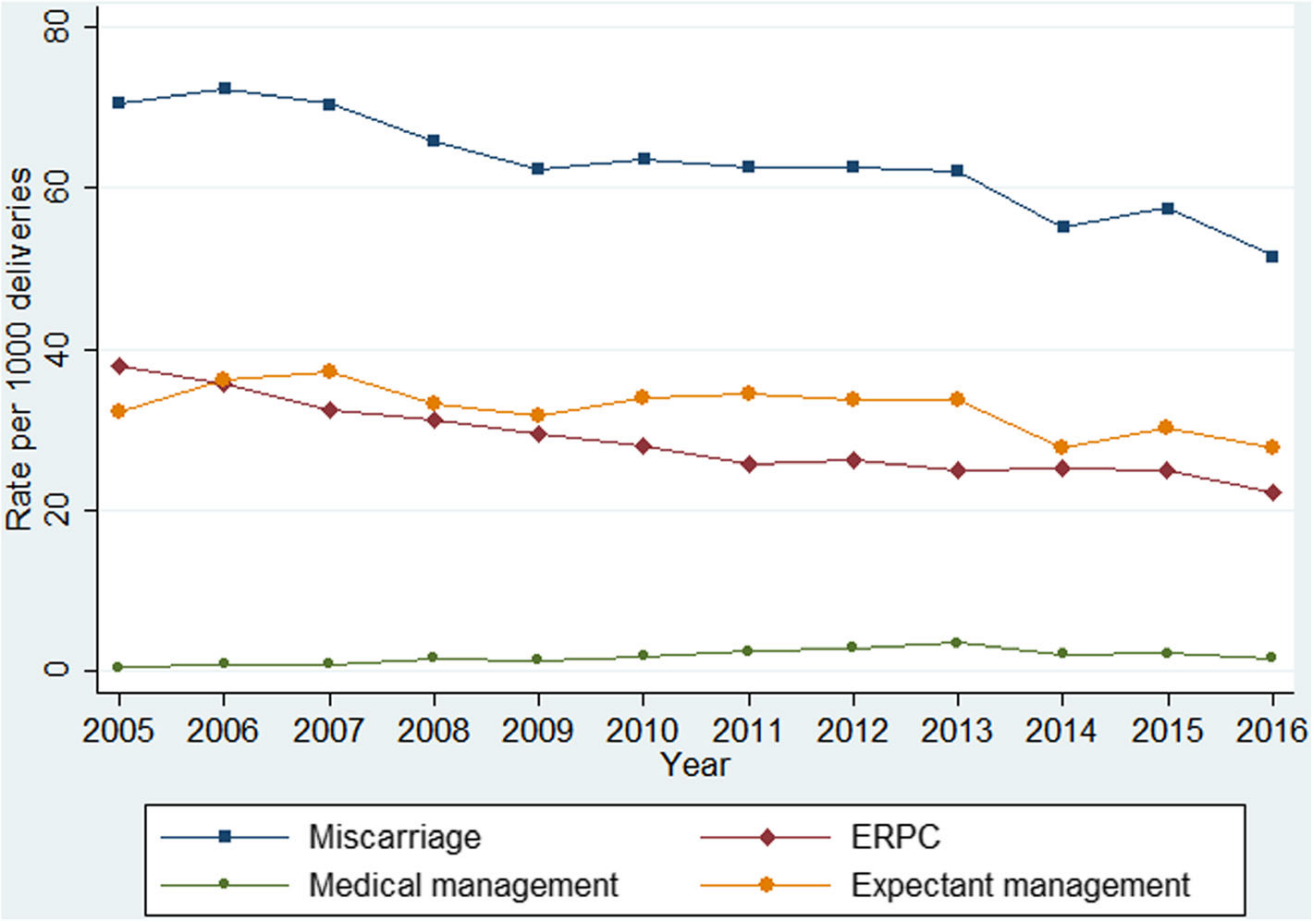
National hospitalisation rates for early miscarriage and type of management

The rates for women with early miscarriage decreased from 70.6/1000 deliveries (95% CI 68.4 to 72.8) in 2005 to an incidence rate of 51.5/1000 deliveries (95% CI 49.7 to 53.3) in 2016 (Fig. 1). The risk of being hospitalised for early miscarriage increased steadily with age, with the exception of women aged between 25 to 29 years old, who had a lower risk (adjusted incidence rate ratio 0.61; 95% CI 0.59 to 0.63). Women of 40 years of age or older had approximately a three-fold increaded risk of being hospitalised than women younger than 25 years old (adjusted incidence rate ratio 3.34; 95% CI 3.22 to 3.45). Public patients had almost double the risk of being hospitalised compared to private patients (Table 1).

**Fig. 2:**
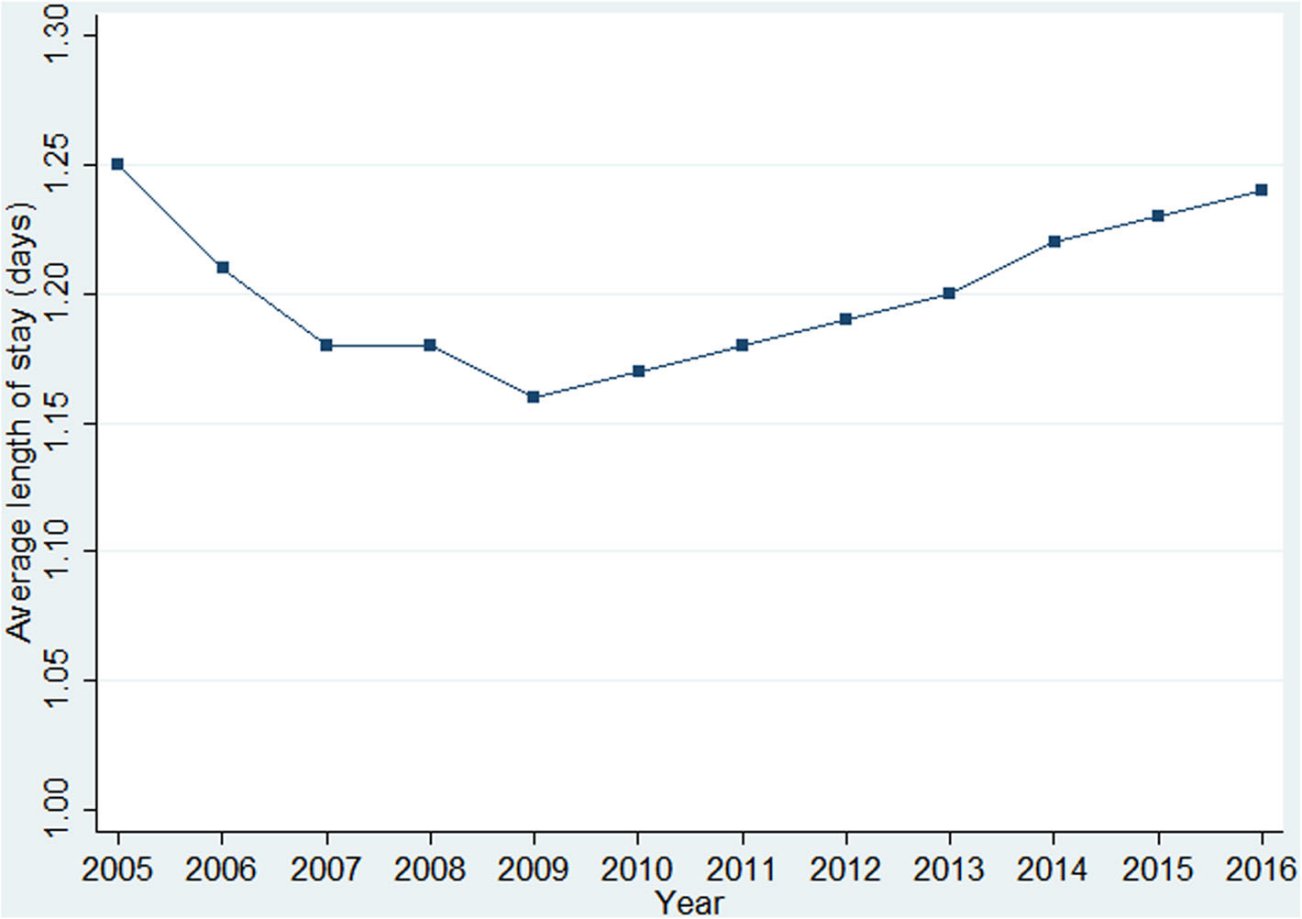
Average length of stay (days) of hospitalisations for early miscarriage

Among hospitalisations for early miscarriage over the same time period, evacuation of retained products of conception was undertaken in almost half of the total sample (*n* = 22,897; 45.3%), and only 2.8% were medically managed (*n* = 1404). Half of the women were expectantly managed (*n* = 26,225; 51.9%); among those, only 3.5% had other type of treatment (*n* = 914). Expectant management remained the most frequent type of treatment over the study period (Fig. 1). Evacuation of retained products of conception gradually decreased from 38.0/1000 deliveries (95% CI 36.4 to 39.6) in 2005 to 22.3/1000 deliveries (95% CI 21.1 to 23.5) in 2016. Medical management steadily increased over time from 0.4/1000 deliveries (95% CI 0.2 to 0.5) in 2005 to 1.6/1000 deliveries (95% CI 1.3 to 1.9) in 2016 (Fig. 1). The average length of stay for early miscarriage fluctuated during the 12-year period from 1.3 days (SD 0.8) in 2005 to 1.2 (SD 0.7) days in 2016; with an overall average of 1.2 days (SD 0.7) (Fig. 2). Approximately 86.4% (*n* = 43,679) of inpatients for early miscarriage stayed in hospital for 1 day and 10.0% (*n* = 5049) stayed for 2 days, with only 3.6% (*n* = 1810) having a length of stay of more than 2 days.

Among the 50,538 hospitalisations for early miscarriage, 554 (1.1%) had a blood transfusion and 1810 (3.6%) had a length of stay longer than 2 days (Table 2). The risk of blood transfusion among hospitalisations for early miscarriage increased over time. No significant differences were found for maternal age and risk of blood transfusion, except for those women who were 25 to 29 years old compared to those younger than 25 years old. Public patients had more than double the risk of a blood transfusion than private patients (adjusted incidence rate ratio 2.5; 95% CI 1.9 to 3.3). Women who were medically, expectantly treated or who had another type of treatment had less blood transfusions as those undergoing evacuation of retained products of conception (adjusted incidence rate ratio 0.3; CI 0.1 to 0.5 & adjusted incidence rate ratio 0.3; 95% CI 0.2 to 0.4 respectively). Incomplete miscarriage had almost two times the rate of blood transfusion compared to early miscarriage (adjusted incidence rate ratio 1.5; 95% CI 1.1 to 2.0; Table 2).

**Table 2:** Blood transfusion and length of stay over 2 days for hospitalisations of early miscarriage, 2005–2016

		Blood Transfusion				Length of stay over 2 days			
	No of hospitalisations for early miscarriage	*n*	%	Crude incidence rate ratio (95% CI)	Adjusted incidence rate ratio ^a^ (95% CI)	*n*	%	Crude incidence rate ratio (95% CI)	Adjusted incidence rate ratio ^a^ (95% CI)
All	50,538	554	1.1			1810	3.6		
Year									
2005–2008	17,958	143	0.8	1.0 (ref. group)	1.0 (ref. group)	645	3.6	1.0 (ref. group)	1.0 (ref. group)
2009–2012	17,956	182	1.0	1.3 (1.0–1.6)	1.3 (1.1–1.7)	555	3.1	0.9 (0.8–1.0)	0.9 (0.8–1.0)
2013–2016	14,624	229	1.6	2.0 (1.6–2.4)	2.0 (1.6–2.4)	610	4.2	1.2 (1.0–1.3)	1.2 (1.0–1.3)
Maternal age									
< 25	6404	75	1.2	1.0 (ref. group)	1.0 (ref. group)	310	4.8	1.0 (ref. group)	1.0 (ref. group)
25–29	9071	78	0.9	0.7 (0.5–1.0)	0.7 (0.5–1.0)	350	3.9	0.8 (0.7–0.9)	0.9 (0.7–0.9)
30–34	14,697	154	1.0	0.9 (0.7–1.2)	0.8 (0.6–1.1)	469	3.2	0.7 (0.6–0.8)	0.7 (0.6–0.8)
35–39	14,250	166	1.2	1.0 (0.8–1.3)	0.9 (0.7–1.2)	465	3.3	0.7 (0.6–0.8)	0.7 (0.6–0.8)
40+	6116	81	1.3	1.1 (0.8–1.5)	1.0 (0.7–1.4)	216	3.5	0.7 (0.6–0.9)	0.8 (0.7–0.9)
Health insurance									
Private	8951	56	0.6	1.0 (ref. group)	1.0 (ref. group)	200	2.2	1.0 (ref. group)	1.0 (ref. group)
Public	41,587	498	1.2	1.9 (1.5–2.5)	2.5 (1.9–3.3)	1610	3.9	1.7 (1.5–2.0)	1.7 (1.5–2.0)
Management									
ERPC^b^	22,897	427	1.9	1.0 (ref. group)	1.0 (ref. group)	870	3.8	1.0 (ref. group)	1.0 (ref. group)
Medical treatment	1404	8	0.6	0.3 (0.2–0.6)	0.3 (0.1–0.5)	90	6.4	1.7 (1.4–2.1)	1.5 (1.2–1.9)
Expectant/other treatment	26,225	119	0.5	0.2 (0.2–0.3)	0.3 (0.2–0.4)	850	3.2	0.9 (0.8–0.9)	0.7 (0.6–0.8)
Type of early miscarriage									
Complete	20,700	89	0.4	1.0 (ref. group)	1.0 (ref. group)	718	3.5	1.0 (ref. group)	1.0 (ref. group)
Incomplete	29,835	464	1.6	3.6 (2.9–4.5)	1.5 (1.1–2.0)	1092	3.7	1.1 (1.0–1.2)	0.8 (0.7–1.0)

^a^Adjusted incidence rate ratio from multivariable analysis including all variables in the table; ^b^*ERP*C Evacuation of retained products of conception

The incidence rate ratio for length of stay over 2 days among hospitalisations for early miscarriage was reduced from 2009 to 2012 compared to 2005–2008 and was increased from 2013 to 2016 compared to 2005–2008 (Table 2). The risk of a prolonged stay at the hospital was reduced with advanced maternal age. Public patients had almost twice the risk of having a length of stay over 2 days than private patients (adjusted incidence rate ratio 1.7; 95% CI 1.5 to 1.9; Table 2). Women who were medically treated had almost twice the risk of having a length of stay over 2 days compared to those undergoing evacuation of retained products of conception (adjusted incidence rate ratio 1.5; CI 1.2 to 1.9). Women who were expectantly treated or who had another type of treatment were less likely to have a prolonged stay at the hospital compared to those treated with evacuation of retained products of conception (Table 2). Finally, no significant differences were found between complete and incomplete miscarriage and the risk of an extended stay at the hospital.

## Discussion

This is a population-based study including more than 50 thousand hospitalisations for early miscarriage. The incidence of early miscarriage hospitalisations became 19% less common during 2005–2016, but the risk of blood transfusion doubled. Women aged 40 years or older had a three-fold risk of hospitalisation than those aged 25 years; and public patients had twice the rate. Women undergoing medical management did not have as many blood transfusion as those undergoing evacuation of retained products of conception; whereas it increased the risk of length of stay over 2 days. Incomplete miscarriage was associated with an increased risk of blood transfusion.

It is well-documented that older maternal age is a risk factor for adverse pregnancy outcomes [26–28] and this is further supported by the results in our study. For example,

the maternal and fetal loss cohort study in Denmark also found that women in their late 30s or older had a higher risk of having ectopic pregnancy, miscarriage or stillbirth, irrespective of their reproductive history [28]. We found no other study assessing the possible impact of health insurance coverage on the risk of complications among hospital admissions for early miscarriage. In order to promote a equal provision of care to pregnant women who miscarry in hospital settings, this possible association should be investigated.

It is important to highlight the possible impact of the modification of the ultrasound values used to diagnose early miscarriage. This change was made to reduce false positive cases of early miscarriage (i.e. a patient who may have an early sonogram with unknown viability and another sonogram where fetal heart activity is found) at an international level in 2011, 2012 and 2013 by the Royal College of Obstetrician and Gynaecologists [29], the UK National Institute for Health and Care Excellence [30] and the American College of Radiology [31] respectively. As in other countries, the Royal College of Physicians of Ireland also modified their guidelines in 2011 [6, 32]. One of the recommendations was to perform a second ultrasound scan to confirm the diagnosis of miscarriage when pregnancies are under 8 weeks of gestation [6]. Although the rate of hospitalisations was reducing before the guidelines, it is sometimes the case that guidelines are produced after a period of time when clinical practice has already been changing. For example, the reduction of the incidence of early miscarriage during 2005–2011 may suggest an improvement in early miscarriage diagnosis in the years leading up to the revised clinical guidance.

In contrast to our results, studies carried out by The National Institute for Health and Care Excellent found that women with a miscarriage who are managed expectantly have a higher risk of blood transfusion and more days of bleeding compared to those who have surgical treatment [3, 33]. One possible explanation for these divergent results is that pregnant women with severe haemorrhage or pain were excluded from some randomized controlled trials [34–37]. Another explanation could be that we were unable to explore if women who were surgically managed had initially been expectantly or medically treated as an outpatient. For example, it is well-documented that there is a higher risk of bleeding and unplanned intervention after expectant or medical management compared to surgical treatment (4, 38); with medical management failure varying from 10 to 20% [15, 38].

The sample size of our study population is one of the main strength of this study. In addition, the HIPE data are recorded following standardised methods using the ICD-10-AM diagnosis code across all the hospitals [22]. Because single weeks of gestation were not available, analysis were restricted to early miscarriage before 14 completed weeks of gestation. Analysing second trimester miscarriage would have included pregnancy loss up to 25 completed weeks of gestation, resulting in the inclusion of a number of stillbirths rather than miscarriages in our analysis. A limitation of the study is that only inpatient data are available from the HIPE database [22]. As a result this study will under-estimate the overall burden of early miscarriage given the lack of outpatient data available nationally. However, this study will probably not under-estimate the morbidity as all were hospital based. In order to estimate the overall burden of early miscarriage, both outpatient and inpatient cases should be investigated.

In conclusion, maternal age, type of health insurance, type of treatment and incomplete miscarriage significantly affected the risk of blood transfusion and length of stay over 2 days at the hospital after being adjusted by confounders. However, a better understanding of the morbidities associated with early miscarriage hospitalisations is needed to improve management and care provided.

## Additional file

Additional file 1: Diagnosis, procedures and complications codes for miscarriage. (DOCX 13 kb)
