# Supplementary material for: Miscarriage hospitalisations: a national population-based study of incidence and outcomes, 2005–2016
Source: Reprod Health. 2019 May 9;16:51. doi: 10.1186/s12978-019-0720-y (PMC6507132; doi:10.1186/s12978-019-0720-y)
Supplement: Supplementary file 1 — Diagnosis, procedures and complications codes for miscarriage. (DOCX 13 kb) [file 12978_2019_720_MOESM1_ESM.docx]

**ADDITIONAL FILES**

**Additional file 1.** **Diagnosis, procedures and complications codes for miscarriage.**

| **Diagnosis codes** | **ICD-10-AM** | **Diagnosis/procedure field** |
| --- | --- | --- |
| Miscarriage | O03 | Principal diagnosis |
| **Procedures codes for miscarriage** | **ICD-10-AM** | **Procedure field** |
| **Evacuation of retained products of conception (ERPC)** |  |  |
| Dilation and evacuation of uterus (D&E); Suction curettage of uterus; Dilatation and curettage of uterus (D&C) with and without dilatation; Dilation and curettage; D&C for retained products of conception (RPC) following delivery; excludes that with suction curettage. suction curettage; by suction curettage; for retained products of conception following delivery | 3564303;3564003; 3564000/ 3564001; 1656400; 1656401 | Principal procedures |
| **Manual removal of placenta** | 9048200 | Principal procedures |
| **Medical management*** |  |  |
| Insertion of prostaglandin suppository for induction of abortion; Medical induction of labour, prostaglandin | 9046200; 9046501 | Principal procedures |
| Medical induction of labour, oxytocin; other medical induction of labour; excludes that with surgical induction of labour | 9046500; 9046502 | Principal procedures |
| **Expectant/Other management** |  | Principal procedures |
| No procedure |  |  |
| Other type of management |  |  |
| Other type of treatment including intravenous administration of pharmacological agents, passive immunisation with Rh(D) immunoglobulin and other allied health interventions. | 9619709; 9619902-09;9217300; 9555001-14… | Principal procedures |
| **Outcomes measures** | **ICD-10-AM** | **Procedure field** |
| Blood transfusion | 9206000/1370601/ 1370602/1370603/ 9206200 | Additional procedures |
| Length of stay (LOS) over 2 days |  |  |
| *No specific code of medical management using misoprostol or mifepristone for miscarriage were available. | | |
